# *LEAFY COTYLEDONs*: old genes with new roles beyond seed development

**DOI:** 10.12688/f1000research.21180.1

**Published:** 2019-12-27

**Authors:** De Niu, Yuehui He

**Affiliations:** 1 National Key Laboratory of Plant Molecular Genetics & Shanghai Center for Plant Stress Biology, Chinese Academy of Sciences Center for Excellence in Molecular Plant Sciences, Shanghai, China

**Keywords:** Seed development, LEC1, LEC2, FUS3, pioneer transcription factor, embryonic reprogramming, vernalization, FLC

## Abstract

Seed development is a complex process and consists of two phases: embryo morphogenesis and seed maturation. LEAFY COTYLEDON (LEC) transcription factors, first discovered in *Arabidopsis thaliana* several decades ago, are master regulators of seed development. Here, we first summarize molecular genetic mechanisms underlying the control of embryogenesis and seed maturation by *LEC*s and then provide a brief review of recent findings in the role of *LEC*s in embryonic resetting of the parental ‘memory of winter cold’ in Arabidopsis. In addition, we discuss various chromatin-based mechanisms underlying developmental silencing of *LEC *genes throughout the post-embryonic development to terminate the embryonic developmental program.

## Introduction

Plant seeds are composed mainly of three distinct compartments: embryo, endosperm, and seed coat. Seed development is a complex and critical stage in the life cycle of higher plants. This development process is classified generally into two different phases: a morphogenesis phase, which is initiated after fertilization and consists of cell division and differentiation, apical–basal plant axis establishment, and organogenesis, and a maturation phase, which includes organ expansion, storage macromolecule accumulation, and acquisition of desiccation tolerance ^1, 2^. Each stage of seed development is regulated by a number of transcription factors (TFs), among which are LEAFY COTYLEDON ( *LEC)1*, *LEC2*, and *FUSCA3 *( *FUS3*) ^2^. These three genes, first identified in Arabidopsis, are master transcriptional regulators in seed development ^1, 2^. Loss-of-function mutations in *LEC1*, *LEC2*, or *FUS3* lead to a partial loss of embryo identity and give rise to a homeotic ‘leafy cotyledon’ phenotype: embryos with cotyledons characteristic of leaf traits (for example, trichrome development and anthocyanin accumulation) ^1, 2^. When ectopically expressed during vegetative phases, these genes can induce somatic embryogenesis. Hence, these genes constitute the group of *LEC* genes and appear to be evolutionarily conserved across angiosperms to control seed development from embryogenesis through seed maturation ^2, 3^.

## Old story: *LEC* genes are master regulators of seed development

*LEC1* encodes a subunit of a trimeric nuclear factor Y (NF-Y) TF and its expression is *de novo* activated shortly after fertilization ^4^. Both *LEC2* and *FUS3* encode plant-specific B3-domain TFs and their expression in early embryos is *de novo* activated within 2 and 3 days after fertilization (DAFs), respectively ^4– 6^. *LEC1* partly promotes the expression of both *LEC2* and *FUS3* ^1, 2^. These three master TFs regulate thousands of genes from early through late stages during seed development in Arabidopsis ^7– 9^ and function in partial redundancy as well as synergistically to control seed development ^1, 2^.

The LEC1-bearing heterotrimeric NF-Y, through a DNA-binding subunit (NF-YA), binds the CCAAT motif of the target gene to regulate their expression ^7^. Various genetic and molecular analyses reveal that *LEC1* is a central regulator of seed development and controls embryonic morphogenesis such as to maintain suspensor identity and specifying cotyledon identity and seed maturation, including biosynthesis of storage macromolecules, acquiring desiccation tolerance, and seed dormancy ^1, 2^. Recent studies show that *LEC1* sequentially regulates distinct sets of genes involved in seed development from early through late stages by coordinating with distinctive TFs and hormones at different stages ^7^. In early embryogenesis, *LEC1* directly regulates the expression of the HD-ZPIII TFs *PHAVOLUTA* and *PHABULOSA* (functioning as apical fate master regulators), and the basic leucine zipper (bZIP) TF *SCARECROW* that controls root architecture ^7, 10, 11^, to promote the establishment of the apical–basal plant axis in embryogenesis. In addition, upon a high level of GA accumulation and consequent degradation of the DELLA protein (a LEC1 partner), LEC1 is released to activate the expression of the auxin-biosynthesis genes, including *YUC4* and *YUC10*, leading to auxin accumulation to facilitate embryo morphogenesis ^12^. In the embryo/seed maturation phase, for instance, *LEC1* activates the expression of *CRUCIFERIN* (encoding a seed storage protein) and *FATTY ACID DESTATURASE3*, leading to the accumulation of seed storage macromolecules ^13, 14^.

Apart from the CCAAT motif, recent genome-wide analyses of LEC1 occupancy reveal that other *cis*-regulatory elements are enriched in *LEC1* target genes in seed development, including RY (CATGCA), ABRE [(C/G/T) ACGTG(G/T)(A/C)] and G-box (CACGTG) ^7, 15^. This indicates that LEC1 may partner with other DNA-binding proteins to regulate seed gene expression. It has recently been shown that LEC1 can interact with LEC2 to form a ternary complex that regulates seed gene expression ^16, 17^; in addition, LEC2 and FUS3 can form a heterodimer ^18^. The DNA-binding B3 domains in both LEC2 and FUS3 recognize the RY motif or its variants ^8, 9, 19^; hence, it is not surprising that the RY motif or variants are enriched in LEC1-binding sites. Consistent with similar seed phenotypes in *lec1*, *lec2*, and *fus3*, these three genes regulate common sets of genes that are involved in embryogenesis or seed maturation or both ^7, 8^. Nevertheless, these three genes each have distinct targets in seed development ^7– 9^. Thus, these genes have partially overlapping as well as synergistic functions in the control of seed development.

## New function: *LEC* genes reset the parental ‘memory of winter cold’ in early embryogenesis

Many over-wintering plants in temperate climates acquire competence to flower in spring after experiencing winter cold (prolonged cold exposure) through a process known as vernalization ^20, 21^. The vernalization pathways shut down the expression of a potent floral repressor and this vernalization-mediated repression or ‘vernalized state’ is maintained in cell divisions during subsequent growth and development when the temperature rises in spring, namely epigenetic ‘memory of winter cold’, enabling plants to flower in spring ^21, 22^. However, the ‘memory of winter cold’ must be reset/erased in the next generation to ensure that each generation or growth cycle experiences winter cold prior to flowering and thus flowers at a right season to maximize reproductive success ^22, 23^. In crucifers such as *Arabidopsis thaliana*, the vernalization pathway represses the expression of the potent floral repressor *FLOWERING LOCUS C* ( *FLC*) to enable spring flowering ^21^. Recent studies reveal that shortly after fertilization *LEC1* functions as a pioneer TF to initiate *FLC* resetting/re-activation and that *LEC2* and *FUS3* subsequently function together with *LEC1* to fully re-activate *FLC* expression in early embryogenesis ^19, 24^. Hence, these three *LEC* genes act to reset the parental ‘memory of winter cold’ during early embryogenesis in Arabidopsis.

In Arabidopsis winter annuals, *FLC*, encoding a MADS-box TF, is upregulated by *FRIGIDA* ( *FRI*) to a high level to inhibit precocious flowering in young seedlings prior to winter cold exposure ^25, 26^. FRI, a plant-specific scaffold protein, is enriched at the *FLC* locus and functions as ‘molecular glue’ to recruit active chromatin modifiers such as the histone 3 lysine 4 (H3K4) methyltransferase complex COMPASS-like and the histone 3 lysine 36 (H3K36) methyltransferase EARLY FLOWERING IN SHORT DAYS (EFS, also called as SDG8) and histone acetyltransferases ^27– 29^. This leads to the establishment of an active chromatin environment with multiple active chromatin marks, including H3K4me3, H3K36me3, and histone acetylation, resulting in a high level of *FLC* expression ^29^. When the temperature drops in winter, two homologous B3-bearing proteins VP1/ABI3-LIKE 1 (VAL1) and VAL2 are specifically enriched at a 47–base pair *cis*-regulatory element encompassing two canonical RY motifs (known as ‘cold memory element’ or CME), located near the 5’ end of the first intron of *FLC*, to mediate *FLC* silencing by vernalization ^30, 31^. VAL proteins further recruit Polycomb group (PcG) proteins such as Polycomb repressive complex 2 (PRC2, an H3K27 methyltransferase complex) that deposits the repressive histone mark H3K27 trimethylation (H3K27me3), leading to a silenced chromatin state at *FLC* and consequent *FLC* silencing by vernalization ^31^. After return to warmth, the silenced *FLC* chromatin is stably maintained or ‘memorized’ through cell divisions during subsequent growth and development through DNA replication-coupled H3K27 trimethylation by PRC2 ^32, 33^.

The epigenetic ‘memory of winter cold’ is reset in the next generation. A recent study has revealed that a LEC1-bearing NF-Y (LEC1 NF-Y) pioneer TF initiates *FLC* re-activation/resetting shortly after fertilization ^24^. Loss of *LEC1* function disables *FLC* resetting in early embryogenesis and thus *FLC* remained silenced in the next generation in the absence of winter cold exposure. LEC1 NF-Y binds to CCAAT motifs located in a distal *FLC* 5’ promoter region, enabling the CME to be accessible to LEC2 and FUS3 ^19^. The B3 domains of LEC2, FUS3, VAL1, and VAL2 bind to CME in an identical manner ^19^. In early embryogenesis, LEC2 and FUS3 are progressively enriched at the CME region, whereas the levels of VAL proteins at CME are progressively reduced, resulting in a disruption of PcG-mediated silencing at *FLC* inherited from the vernalized parents ^19^. On the other hand, LEC2 and FUS3 further recruit FRI in complex with active chromatin modifiers to establish an active *FLC* chromatin state, resulting in embryonic *FLC* re-activation/resetting ^19^. The active embryonic *FLC* chromatin state can be transmitted to young seedlings upon seed germination and a high-level expression of *FLC* prevents precocious flowering prior to winter cold exposure ^24^.

## Developmental silencing of *LEC* genes throughout post-embryonic development

*LEC* genes are specifically or primarily expressed in seed development and silenced throughout post-embryonic development under normal growth conditions ^2, 4^. This terminates the embryonic developmental program. Following seed germination, plants enter vegetative growth and development. Furthermore, the silencing of *LEC*s at seedling stages enables VAL1 and VAL2 to bind to CME at the *FLC* locus to mediate *FLC* silencing again by vernalization when winter cold comes ^19^, ensuring that each generation acquires competence to flower after experiencing winter.

*LEC* genes are silenced by chromatin-mediated mechanisms through repressive chromatin modifications throughout post-embryonic development ^2^. Following seed germination, histone deacetylases such as HDA6 and HDA19 are enriched at the *LEC* loci for histone deacetylation ^34, 35^. In addition, Polycomb repressive complex 1 (PRC1), containing an E3 ubiquitin ligase AtBMI1A/AtBMI1B, is enriched at *LEC* loci to mediate histone H2A monoubiquitination (H2Aub), and after H2Aub marking, an H3K27 methyltransferase complex, PRC2, is recruited to deposit the repressive H3K27me3 for transcriptional repression at the *LEC* loci ^36– 40^. In addition to the H2Aub marking, *cis*-regulatory DNA elements, Polycomb responsive elements (PREs), are required for PRC2 recruitment to both *LEC2* and *FUS3* chromatin ^2, 41– 43^; moreover, H3K27 trimethylation at *LEC*s requires ATP-dependent nucleosome remodeling by PICKLE ^44^. The H3K27me3 mark at the *LEC* loci is read by LIKE HETEROCHROMATIN PROTEIN 1 (LHP1) and two Bromo adjacent homology (BAH) domain-bearing proteins known as EARLY BOLTING IN SHORT DAYS (EBS) and SHORT LIFE (SHL) ^39, 45^. These readers further associate with the plant-specific EMBRYONIC FLOWER 1 (EMF1) that mediates chromatin compaction for transcriptional repression ^45– 47^. In short, PRC1-mediated H2A monoubiquitination initiates the repression of *LEC*s, followed by H3K27me3 deposition by PRC2 to establish stable silencing of *LEC*s. This results in the switch from embryonic to post-germinative growth and development and thus termination of the embryonic developmental program.

## Perspectives

*LEC1*, *LEC2*, and *FUS3* play essential roles to regulate seed development from early through late stages. Although there is considerable understanding of the regulatory network of *LEC*s, important questions remain to be answered. For example, how LECs and other transcriptional regulators function together to sequentially regulate various aspects of seed development at different stages. How is the expression of each *LEC* gene *de novo* activated in early embryogenesis? Within hours after fertilization, the expression of *LEC1*, functioning as a pioneer TF, is activated, but the underlying molecular and chromatin mechanisms are essentially unknown.

Plants are sessile and must endure diverse environmental challenges (for example, winter cold) by reprograming transcriptional circuitries typically through chromatin modifications. At certain loci, some environment-induced chromatin marks are heritable through cell divisions after relief from environmental inputs. In addition to resetting/erasing of the winter cold–induced H3K27me3 at *FLC*, do *LEC* genes function to erase H3K27me3 at other loci and/or other chromatin marks in the Arabidopsis genome in early embryogenesis? Seeds are essential resources of nutrients for humans and animals. Addressing these and related questions will advance the molecular, genetic, and epigenetic understanding of seed development, providing effective strategies for genetic manipulation of seed development toward high yield and better nutrients.

## Abbreviations

CME, cold memory element; FLC, FLOWERING LOCUS C; FRI, FRIGIDA; FUS3, FUSCA3; H2Aub, histone H2A monoubiquitination; H3K27me3, histone 3 lysine-27 trimethylation; LEC1, LEAFY COTYLEDON 1; LEC2, LEAFY COTYLEDON 2; LEC, LEAFY COTYLEDON; NF-Y, nuclear factor Y; PcG, Polycomb group; PRC1, Polycomb repressive complex 1; PRC2, Polycomb repressive complex 2; TF, transcription factor; VAL1, VIVIPAROUS/ABI3-LIKE 1; VAL2, VIVIPAROUS/ABI3-LIKE 2

## References

[1] Jo L Pelletier JM Harada JJ: Central role of the LEAFY COTYLEDON1 transcription factor in seed development. *J Integr Plant Biol.* 2019;61(5):564–80. 10.1111/jipb.12806 30916433

[2] Lepiniec L Devic M Roscoe TJ: Molecular and epigenetic regulations and functions of the LAFL transcriptional regulators that control seed development. *Plant Reprod.* 2018;31(3):291–307. 10.1007/s00497-018-0337-2 29797091

[3] Meinke DW Franzmann LH Nickle TC: Leafy Cotyledon Mutants of Arabidopsis. *Plant Cell.* 1994;6(8):1049–1064. 10.1105/tpc.6.8.1049 12244265 PMC160500

[4] Le BH Cheng C Bui AQ: Global analysis of gene activity during *Arabidopsis* seed development and identification of seed-specific transcription factors. *Proc Natl Acad Sci U S A.* 2010;107(18):8063–70. 10.1073/pnas.1003530107 20385809 PMC2889569

[5] Stone SL Kwong LW Yee KM: *LEAFY COTYLEDON2* encodes a B3 domain transcription factor that induces embryo development. *Proc Natl Acad Sci U S A.* 2001;98(20):11806–11. 10.1073/pnas.201413498 11573014 PMC58812

[6] Luerßen H Kirik V Herrmann P: *FUSCA3* encodes a protein with a conserved VP1/AB13-like B3 domain which is of functional importance for the regulation of seed maturation in *Arabidopsis thaliana*. *Plant J.* 1998;15(6):755–64. 10.1046/j.1365-313x.1998.00259.x 9807814

[7] Pelletier JM Kwong RW Park S: LEC1 sequentially regulates the transcription of genes involved in diverse developmental processes during seed development. *Proc Natl Acad Sci U S A.* 2017;114(32):E6710–E6719. 10.1073/pnas.1707957114 28739919 PMC5559047

[8] Wang F Perry SE: Identification of direct targets of FUSCA3, a key regulator of Arabidopsis seed development. *Plant Physiol.* 2013;161(3):1251–64. 10.1104/pp.112.212282 23314941 PMC3585594

[9] Braybrook SA Stone SL Park S: Genes directly regulated by LEAFY COTYLEDON2 provide insight into the control of embryo maturation and somatic embryogenesis. *Proc Natl Acad Sci U S A.* 2006;103(9):3468–73. 10.1073/pnas.0511331103 16492731 PMC1413938

[10] Smith ZR Long JA: Control of Arabidopsis apical-basal embryo polarity by antagonistic transcription factors. *Nature.* 2010;464(7287):423–6. 10.1038/nature08843 20190735 PMC2841697

[11] Di Laurenzio L Wysocka-Diller J Malamy JE: The SCARECROW gene regulates an asymmetric cell division that is essential for generating the radial organization of the Arabidopsis root. *Cell.* 1996;86(3):423–33. 10.1016/s0092-8674(00)80115-4 8756724

[12] Hu Y Zhou L Huang M: Gibberellins play an essential role in late embryogenesis of *Arabidopsis*. *Nat Plants.* 2018;4(5):289–98. 10.1038/s41477-018-0143-8 29725104

[13] Yamamoto A Kagaya Y Toyoshima R: Arabidopsis NF-YB subunits LEC1 and LEC1-LIKE activate transcription by interacting with seed-specific ABRE-binding factors. *Plant J.* 2009;58(5):843–56. 10.1111/j.1365-313X.2009.03817.x 19207209

[14] Mendes A Kelly AA van Erp H: bZIP67 regulates the omega-3 fatty acid content of *Arabidopsis* seed oil by activating *fatty acid desaturase3*. *Plant Cell.* 2013;25(8):3104–16. 10.1105/tpc.113.116343 23995083 PMC3784602

[15] Junker A Mönke G Rutten T: Elongation-related functions of LEAFY COTYLEDON1 during the development of *Arabidopsis thaliana*. *Plant J.* 2012;71(3):427–42. 10.1111/j.1365-313X.2012.04999.x 22429691

[16] Baud S Kelemen Z Thévenin J: Deciphering the Molecular Mechanisms Underpinning the Transcriptional Control of Gene Expression by Master Transcriptional Regulators in Arabidopsis Seed. *Plant Physiol.* 2016;171(2):1099–112. 10.1104/pp.16.00034 27208266 PMC4902591

[17] Boulard C Thévenin J Tranquet O: LEC1 (NF-YB9) directly interacts with LEC2 to control gene expression in seed. *Biochim Biophys Acta Gene Regul Mech.* 2018;1861(5):443–50. 10.1016/j.bbagrm.2018.03.005 29580949

[18] Tang LP Zhou C Wang SS: FUSCA3 interacting with LEAFY COTYLEDON2 controls lateral root formation through regulating *YUCCA4* gene expression in *Arabidopsis thaliana*. *New Phytol.* 2017;213(4):1740–54. 10.1111/nph.14313 27878992

[19] Tao Z Hu H Luo X: Embryonic resetting of the parental vernalized state by two B3 domain transcription factors in Arabidopsis. *Nat Plants.* 2019;5(4):424–35. 10.1038/s41477-019-0402-3 30962525

[20] Xu S Chong K: Remembering winter through vernalisation. *Nat Plants.* 2018;4(12):997–1009. 10.1038/s41477-018-0301-z 30478363

[21] Bouché F Woods DP Amasino RM: Winter Memory throughout the Plant Kingdom: Different Paths to Flowering. *Plant Physiol.* 2017;173(1):27–35. 10.1104/pp.16.01322 27756819 PMC5210730

[22] Andrés F Coupland G: The genetic basis of flowering responses to seasonal cues. *Nat Rev Genet.* 2012;13(9):627–39. 10.1038/nrg3291 22898651

[23] He Y Li Z: Epigenetic Environmental Memories in Plants: Establishment, Maintenance, and Reprogramming. *Trends Genet.* 2018;34(11):856–66. 10.1016/j.tig.2018.07.006 30144941

[24] Tao Z Shen L Gu X: Embryonic epigenetic reprogramming by a pioneer transcription factor in plants. *Nature.* 2017;551(7678):124–8. 10.1038/nature24300 29072296

[25] Michaels SD Amasino RM: Loss of *FLOWERING LOCUS C* activity eliminates the late-flowering phenotype of *FRIGIDA* and autonomous pathway mutations but not responsiveness to vernalization. *Plant Cell.* 2001;13(4):935–41. 10.1105/tpc.13.4.935 11283346 PMC135534

[26] Sheldon CC Burn JE Perez PP: The *FLF* MADS box gene: a repressor of flowering in Arabidopsis regulated by vernalization and methylation. *Plant Cell.* 1999;11(3):445–58. 10.1105/tpc.11.3.445 10072403 PMC144185

[27] Choi K Kim J Hwang HJ: The *FRIGIDA* complex activates transcription of *FLC*, a strong flowering repressor in *Arabidopsis*, by recruiting chromatin modification factors. *Plant Cell.* 2012;23(1):289–303. 10.1105/tpc.110.075911 21282526 PMC3051252

[28] Ko JH Mitina I Tamada Y: Growth habit determination by the balance of histone methylation activities in *Arabidopsis*. *EMBO J.* 2010;29(18):3208–15. 10.1038/emboj.2010.198 20711170 PMC2944069

[29] Li Z Jiang D He Y: FRIGIDA establishes a local chromosomal environment for FLOWERING LOCUS C mRNA production. *Nat Plants.* 2018;4(10):836–46. 10.1038/s41477-018-0250-6 30224662

[30] Qüesta JI Song J Geraldo N: *Arabidopsis* transcriptional repressor VAL1 triggers Polycomb silencing at *FLC* during vernalization. *Science.* 2016;353(6298):485–8. 10.1126/science.aaf7354 27471304

[31] Yuan W Luo X Li Z: A *cis* cold memory element and a *trans* epigenome reader mediate Polycomb silencing of *FLC* by vernalization in *Arabidopsis*. *Nat Genet.* 2016;48(12):1527–34. 10.1038/ng.3712 27819666

[32] Jiang D Berger F: DNA replication-coupled histone modification maintains Polycomb gene silencing in plants. *Science.* 2017;357(6356):1146–9. 10.1126/science.aan4965 28818970

[33] Yang H Berry S Olsson TSG: Distinct phases of Polycomb silencing to hold epigenetic memory of cold in *Arabidopsis*. *Science.* 2017;357(6356):1142–5. 10.1126/science.aan1121 28818969

[34] Zhou Y Tan B Luo M: HISTONE DEACETYLASE19 interacts with HSL1 and participates in the repression of seed maturation genes in *Arabidopsis* seedlings. *Plant Cell.* 2013;25(1):134–48. 10.1105/tpc.112.096313 23362207 PMC3584530

[35] Chhun T Chong SY Park BS: HSI2 Repressor Recruits MED13 and HDA6 to Down-Regulate Seed Maturation Gene Expression Directly During Arabidopsis Early Seedling Growth. *Plant Cell Physiol.* 2016;57(8):1689–706. 10.1093/pcp/pcw095 27335347

[36] Yang C Bratzel F Hohmann N: VAL- and AtBMI1-mediated H2Aub initiate the switch from embryonic to postgerminative growth in *Arabidopsis*. *Curr Biol.* 2013;23(14):1324–9. 10.1016/j.cub.2013.05.050 23810531

[37] Wang H Liu C Cheng J: Arabidopsis Flower and Embryo Developmental Genes are Repressed in Seedlings by Different Combinations of Polycomb Group Proteins in Association with Distinct Sets of Cis-regulatory Elements. *PLoS Genet.* 2016;12(1):e1005771. 10.1371/journal.pgen.1005771 26760036 PMC4711971

[38] Molitor AM Bu Z Yu Y: *Arabidopsis* AL PHD-PRC1 complexes promote seed germination through H3K4me3-to-H3K27me3 chromatin state switch in repression of seed developmental genes. *PLoS Genet.* 2014;10(1):e1004091. 10.1371/journal.pgen.1004091 24465219 PMC3900384

[39] Bratzel F López-Torrejón G Koch M: Keeping cell identity in *Arabidopsis* requires PRC1 RING-finger homologs that catalyze H2A monoubiquitination. *Curr Biol.* 2010;20(20):1853–9. 10.1016/j.cub.2010.09.046 20933424

[40] Merini W Romero-Campero FJ Gomez-Zambrano A: The Arabidopsis Polycomb Repressive Complex 1 (PRC1) Components AtBMI1A, B, and C Impact Gene Networks throughout All Stages of Plant Development. *Plant Physiol.* 2017;173(1):627–41. 10.1104/pp.16.01259 27837089 PMC5210725

[41] Berger N Dubreucq B Roudier F: Transcriptional regulation of *Arabidopsis LEAFY COTYLEDON2* involves *RLE*, a *cis*-element that regulates trimethylation of histone H3 at lysine-27. *Plant Cell.* 2011;23(11):4065–78. 10.1105/tpc.111.087866 22080598 PMC3246333

[42] Xiao J Jin R Yu X: *Cis* and *trans* determinants of epigenetic silencing by Polycomb repressive complex 2 in *Arabidopsis*. *Nat Genet.* 2017;49(10):1546–52. 10.1038/ng.3937 28825728

[43] Roscoe TJ Vaissayre V Paszkiewicz G: Regulation of *FUSCA3* Expression During Seed Development in Arabidopsis. *Plant Cell Physiol.* 2019;60(2):476–87. 10.1093/pcp/pcy224 30462310

[44] Zhang H Bishop B Ringenberg W: The CHD3 remodeler PICKLE associates with genes enriched for trimethylation of histone H3 lysine 27. *Plant Physiol.* 2012;159(1):418–32. 10.1104/pp.112.194878 22452853 PMC3375975

[45] Li Z Fu X Wang Y: Polycomb-mediated gene silencing by the BAH-EMF1 complex in plants. *Nat Genet.* 2018;50(9):1254–61. 10.1038/s41588-018-0190-0 30082786

[46] Wang Y Gu X Yuan W: Photoperiodic control of the floral transition through a distinct polycomb repressive complex. *Dev Cell.* 2014;28(6):727–36. 10.1016/j.devcel.2014.01.029 24613395

[47] Calonje M Sanchez R Chen L: EMBRYONIC FLOWER1 participates in polycomb group-mediated *AG* gene silencing in *Arabidopsis*. *Plant Cell.* 2008;20(2):277–91. 10.1105/tpc.106.049957 18281509 PMC2276442

